# Rural–urban differences in the association between home-based community care services satisfaction and mental health status among older adults in Zhejiang Province, China: a cross-sectional study

**DOI:** 10.3389/fpubh.2024.1449670

**Published:** 2024-11-29

**Authors:** Yuchen Ying, Lifang Dong, Li Zhang, Fanqian Kong, Jiani Yang, Xiaoling Huang

**Affiliations:** ^1^School of Health Services and Healthcare, Ningbo College of Health Sciences, Ningbo, Zhejiang, China; ^2^Department of Psychosomatic Medicine, The First Affiliated Hospital of Ningbo University, Ningbo, Zhejiang, China; ^3^School of Nursing, Ningbo College of Health Sciences, Ningbo, Zhejiang, China; ^4^Youth League Committee, Ningbo College of Health Sciences, Ningbo, Zhejiang, China; ^5^Department of Medical Record and Statistics, Ningbo Medical Center Lihuili Hospital, Ningbo, Zhejiang, China; ^6^Department of Development and Planning, Ningbo College of Health Sciences, Ningbo, Zhejiang, China

**Keywords:** home-based community care service, mental health status, Chinese older adults, cross-sectional study, rural–urban differences

## Abstract

**Objective:**

This study aims to examine the association between home-based community care services (HBCCS) and mental health in older adults and specifically analyzes rural–urban differences in the association.

**Methods:**

This cross-sectional study enrolled 852 older adults from Zhejiang Province, China. The Patient Health Questionnaire (PHQ-9), 7-item Generalized Anxiety Disorder (GAD-7), University of California Los Angeles 3-item Loneliness Scale (UCLA-3), and the Mental Health (MH) component score of the 36-item Short Form (SF-36) were used to measure self-reported mental health status. Four categories of community care services were examined: daily, medical, social and recreational, and spiritual comfort. Satisfaction with community care services was assessed using self-reported measures. We used a multiple linear regression model.

**Results:**

Satisfaction with daily care services, social and recreational services, and spiritual comfort services in rural older adults was significantly higher than in urban older adults (*p* = 0.016, *p* < 0.001, *p* < 0.001, respectively). Rural older adults reported lower scores on the PHQ-9, GAD-7, and UCLA-3 than urban older adults (*p* < 0.001, *p* = 0.003, *p* = 0.001, respectively) and had significantly higher scores on the SF-36 MH than urban older adults (*p* < 0.001). Among urban older adults, medical care services satisfaction was negatively related to the PHQ-9 and UCLA-3 scores (*β* = −0.296, *p* = 0.004; *β* = −0.447, *p* = 0.009, respectively). A lower UCLA-3 score was associated with higher levels of satisfaction with social and recreational services and with spiritual comfort services (*β* = −0.426, *p* = 0.010; *β* = −0.523, *p* = 0.002, respectively). A higher level of spiritual comfort services satisfaction was associated with a lower SF-36 MH score (*β* = 0.646, *p* < 0.001). Among rural older adults, medical care services satisfaction was negatively related to the GAD-7 score (*β* = −0.327, *p* = 0.028).

**Conclusion:**

Home-based community care services satisfaction was positively associated with older adults’ mental health status in Zhejiang Province. More attention should be paid to maintaining relevant satisfaction with HBCCS to ensure positive mental health among rural and urban older adults.

## 1 Introduction

China has the largest population of older adults worldwide (1). It is predicted that the number of adults over the age of 65 in China will increase to approximately 1.5 billion by 2050, with approximately one in six people expected to be at least 65 years old (2). More than 90% of older adults are living at home (3). Given reduced family support and inadequate medical and old-age care resources, the Chinese government has been promoting the concept of “home-based community care services (HBCCS)” to encourage older adults to obtain higher-quality living conditions at home and promote their health and well-being (4).

Home-based community care services refers to a range of services in the areas of daily living support, healthcare, leisure, and spiritual care (5). In China, HBCCS can be provided by various sources, including government agencies, non-profit organizations, and private entities to meet the needs of Chinese older adults living at home. Depending on the specific services required, HBCCS providers may include medical professionals, social workers, and other trained personnel (6). The Chinese government plays a significant role in providing and regulating HBCCS, and has issued several policy documents to promote the development of HBCCS (7, 8). Satisfaction with HBCCS has become a particularly important issue because older adults often prefer to receive care at home rather than at institutions. Satisfaction with HBCCS mainly refers to the degree to which older adults’ self-perceived care needs are met (9). However, studies on satisfaction with HBCCS in China are limited.

Because most older adults in China live in rural areas, the numbers of older adults in villages are increasing faster than in cities (10). In addition, with the process of modernization and urbanization, there is a clear trend of labor flow from rural areas to cities, resulting in a steady rise in the number of older adults remaining in rural areas (11). Given the link between old-age dependency and rural homes where younger family members have left to go to cities, notable numbers of rural older adults seek HBCCS for successful aging (12).

Increased attention has been paid to improvement of HBCCS for rural older adults, especially in developed regions, such as Zhejiang Province. Zhejiang Province has specifically sought to promote high-quality development and common prosperity, which is important given that common prosperity is a key feature of Chinese-style modernization (13). This goal is possible because the economy of Zhejiang Province is strong and has developed in a balanced manner, advancing prosperity. The incomes of urban and rural residents have ranked first in China for 20 and 36 consecutive years, respectively, and the income gap between urban and rural residents is only 1.96, which is much lower than the national level (2.56) (14).

Chinese older adults are currently experiencing an increased risk of mental health problems (15–17). Mental health distress leads to a higher incidence of disability, decline in quality of life, and increased risk of mortality (18). Studies have revealed that older urban adults in China have better psychological health than rural counterparts (19). Additionally, studies have shown that older Chinese people in rural villages are more vulnerable to loneliness and social isolation, which are linked to higher risks of experiencing mental health problems than for those in urban areas (20).

Nevertheless, while the Chinese government has regarded HBCCS as a crucial means to promote mental health among older adults, few studies have focused on satisfaction with HBCCS, and the association between HBCCS satisfaction and mental health among older adults remains unclear in China. Studies on the association between HBCCS and mental health of older adults have mainly been conducted in developed countries, with differing findings. Some studies have shown that HBCCS significantly improve the mental health of older adults (4, 6), whereas others have found that low-quality care services may aggravate disease symptoms (21). Furthermore, considering the different rural–urban structures and regional economic differences in China, it is essential to compare satisfaction with HBCCS, mental health status, and their correlation among older adults in specific urban and rural backgrounds.

Therefore, this study aimed to explore the association between HBCCS satisfaction and the mental health status of older Chinese adults in Zhejiang Province. Moreover, considering the rural–urban disparity, the results of this study were compared across location groups. We hypothesized that satisfaction with HBCCS would be positively associated with older adults’ mental health status.

## 2 Methods

### 2.1 Study design and sampling

This cross-sectional household study was performed in Zhejiang Province from March to August, 2021. Stratified sampling was used in this study. Participants were recruited from the cities of Hangzhou, Ningbo, Shaoxing, Quzhou, and Lishui, which ranked from low to high in economic development in Zhejiang Province, representing the overall average development level. Five districts (counties) from each city were randomly selected. Subsequently, we randomly and proportionally selected four or five communities from each district (county). The inclusion criteria were communities that (1) provided HBCCS to local older adults, (2) had provided HBCCS for at least 1 month, and (3) did not provide other types of older-care services. Older adults from any of the communities receiving HBCCS were included in this study.

### 2.2 Participants

Participants in this study were older Chinese adults aged 60 years and above. The exclusion criteria were as follows: (1) self-reported history of mental disorders; (2) cognitive impairment with a Mini-Mental State Examination score ≤ 24 (44); (3) substance abuse; (4) hearing disorder or other disabilities (22). All the participants were informed of the study procedure through face-to-face interviews. Informed consent was obtained from each participant or, wherever appropriate, from their primary caregivers. Eligible Participants were asked to administer standardized questionnaires. Overall, 1,162 participants were recruited for this study. Among them, 310 were excluded due to incomplete information. A total of 852 participants completed the questionnaires. The response rate was 73.3%.

### 2.3 Sample size calculation

Based on the population covered by the selected communities included in the study (57,500 older adults aged ≥60 years), for a 95% confidence level and sample error tolerance of 5%, the sample size was determined using the formula (23) *n* = *N*/(1 + *Ne*^2^), where *n* = required sample size, *N* = total population of older adults enrolled in the selected communities (57,500), and *e* = error tolerance (*α* = 0.05). The calculation yielded a sample size of 397. An additional 30% was added to the sample size to account for the inevitable losses in the survey conducted during follow-up, leading to a final sample size of at least 516 individuals assessed at baseline.

### 2.4 Data collection

The questionnaires were administered by a trained research team, which was composed of more than 30 members. The survey was conducted in either a designated private room in the HBCCS center or the participants’ homes. Two researchers helped each participant to complete the questionnaire. One researcher explained the questions to the participants because some older adults were illiterate or unable to understand the questions, and another researcher filled out questionnaires based on the respondents’ answers. Participants had the right to withdraw or interrupt the survey at any time if they felt tired or bored. The order in which the questionnaires were administered was determined at random. All questionnaires were administered anonymously and in accordance with ethical standards. During data collection, we asked permission to digitally record the survey to protect data.

### 2.5 Outcome measures

#### 2.5.1 Dependent variable

The Chinese version of the Patient Health Questionnaire (PHQ-9) was used to assess depressive symptoms, with scores ranging from 0 to 27, and with scores of 5, 10, 15, and 20 indicating mild, moderate, moderately severe, and severe level of depressive symptoms, respectively (24). The PHQ-9 has been widely used in China and various studies have confirmed its reliability and validity (25). The Cronbach’s *α* was 0.83 in this study.

The Chinese version of the 7-item Generalized Anxiety Disorder (GAD-7) scale was used to assess anxiety symptoms, with scores ranging from 0 to 21. Cutoff points of 5, 10, and 15 represent levels of anxiety as being mild, moderate, and severe, respectively (26). The GAD-7 has been widely used in China and its reliability and validity have been confirmed (27). The Cronbach’s *α* was 0.87 in this study.

The Chinese version of the University of California Los Angeles 3-item Loneliness Scale (UCLA-3) was used to assess feelings of loneliness. Scale scores range from 3 to 9, with higher scores representing higher levels of perceived loneliness (28). The Chinese version of UCLA-3 has been used among older Chinese people, with studies confirming its reliability and validity (29). The Cronbach’s *α* was 0.93 in this study.

The 36-item Short-Form Health Survey (SF-36) mental health (MH) dimension score, ranging from 0 to 100, was constructed to measure mental well-being, with higher scores indicating better mental health status (30). The Chinese version of the SF-36 has shown good reliability and validity, with appropriate cultural equivalence among older Chinese individuals (31). The Cronbach’s *α* was 0.85 in this study.

#### 2.5.2 Independent variables

The demographic items included age, sex, number of diseases (0, 1–2, >2), self-reported health status on a 5-point scale ranging from 1 (very bad) to 5 (very good), self-care ability (none, partly, and completely), marital status (currently married/co-habitating, currently single), monthly income (<$155, $155–$310, $310–$465, $465–$620, ≥$620), location (urban, rural), and education level (junior high school and lower, senior high school and higher).

Considering collinearity problems, we separated the available HBCCS into four categories: daily care services, medical care services, social and recreational services, and spiritual comfort services. Daily care services refer to basic life support services provided by the government, neighborhood committees, and communities. Medical care services are basic health-related services provided by community healthcare centers or local clinics. Social and recreational services mainly refer to cultural and entertainment services organized by the community. Spiritual comfort services refer to the social attention and comfort provided in relation to the spiritual needs of older adults (4). Satisfaction with HBCCS was assessed on a 5-point scale, with scores ranging from 1 (very dissatisfied) to 5 (very satisfied) using the question “In general, how do you feel about your daily care/medical care/social and recreational/spiritual comfort services provided by the community?”.

### 2.6 Ethical approval

Ethical approval was obtained from the Ethics Committee of Ningbo First Hospital, Ningbo, China (approval number: 2020-R042), and all procedures were performed in accordance with the principles of the Declaration of Helsinki. The voluntary nature of participation was emphasized throughout the process. The data collection process adhered to the ethical guidelines.

### 2.7 Statistical analysis

The Kolmogorov–Smirnov test was utilized to assess the normal distribution of continuous data. Continuous variables were reported as mean ± standard deviation (*SD*) or median [interquartile range (IQR)], depending on the data distribution. Categorical variables were presented as percentages. Univariate analysis involved Student’ s *t*-test or Mann–Whitney *U*-test for continuous variables, based on the data distribution, and chi-square tests were employed for categorical variables.

To evaluate the association between the quality of HBCCS and the mental health of the older adults, a multiple linear regression model was implemented with adjustments for sex, age, self-reported health status, number of diseases, self-care ability, marital status, monthly income, and education level. A robustness test was conducted to ensure the reliability of the results. In this study, we removed all the control variables to further examine the association between HBCCS satisfaction and the mental health among older adults. All statistical analyses were performed using SPSS version 26.0 (SPSS Inc., Chicago, IL, United States) software, and statistical significance was considered at the alpha level of *p* < 0.05 or *p* < 0.01.

## 3 Results

### 3.1 Descriptive characteristics, HBCCS satisfaction, and mental health

A total of 852 participants were enrolled in this study. The baseline characteristics of the included older adults are presented in Table 1. Regarding residential areas, 468 (54.93%) respondents lived in urban areas, and 384 (45.07%) lived in rural areas. The average age was 77.55 (*SD* = 9.06). Regarding self-reported health status, the rural older adults reported higher average scores than the urban older adults (*p* < 0.001). Satisfaction with daily care, social and recreational, and spiritual comfort services was significantly higher in the rural older adults than in the urban older adults (*p* = 0.016, *p* < 0.001, *p* < 0.001, respectively). Regarding mental health among the respondents, the rural older adults reported lower scores on the PHQ-9, GAD7, and UCLA-3 than the urban older adults (*p* < 0.001, *p* = 0.003, *p* = 0.001, respectively), and had significantly higher scores on the SF-36 MH than the urban older adults (*p* < 0.001).

**Table 1:** The baseline characteristics of the urban and rural older adult participants.

Variable	Total	Urban (*n* = 468)	Rural (*n* = 384)	*p-*value
Age	77.55(9.06)	77.91 ± 9.07	77.11 ± 9.03	0.202
Sex				0.583
Male	386(45.31)	216(46.15)	170(44.27)	
Female	466(54.69)	252(53.85)	214(55.73)	
Self-reported health status	3.82(0.96)	3.72 ± 1.08	3.94 ± 0.77	<0.001
Number of Disease				0.033
0	165(19.37)	78(16.67)	87(22.66)	
1–2	312(36.62)	168(35.90)	144(37.50)	
>2	375(44.01)	222(47.44)	153(39.84)	
Self-care ability				<0.001
None	56(6.57)	50(10.68)	6(1.56)	
Partly	336(39.44)	173(36.97)	163(42.45)	
Completely	460(53.99)	245(52.35)	215(55.99)	
Marital status				<0.001
Currently married/cohabitating	783(91.90)	412(88.03)	371(96.61)	
Currently single	69(8.10)	56(11.97)	13(3.39)	
Monthly income				0.054
<$155	31(3.64)	25(5.34)	6(1.56)	
$155–$310	247(28.99)	130(27.78)	117(30.47)	
$310–$465	144(16.90)	80(17.09)	64(16.67)	
$465–$620	255(29.93)	135(28.85)	120(31.25)	
≥$620	175(20.54)	98(20.94)	77(20.05)	
Education				0.342
Junior high school and lower	388(45.54)	220(47.01)	168(43.75)	
Senior high school and higher	464(54.46)	248(52.99)	216(56.25)	
Satisfaction of community care services				
Daily care	3.72(0.93)	3.65 ± 1.01	3.80 ± 0.80	0.016
Medical care	3.83(0.97)	3.78 ± 1.06	3.89 ± 0.84	0.095
Social and recreational	3.93(0.96)	3.83 ± 1.05	4.05 ± 0.83	<0.001
Spiritual comfort	3.89(0.97)	3.78 ± 1.09	4.03 ± 0.79	<0.001
Mental health				
PHQ-9	4.80(1.86)	5.07 ± 2.22	4.47 ± 1.21	<0.001
GAD-7	5.47(2.63)	5.71 ± 2.85	5.18 ± 2.31	0.003
UCLA Loneliness Scale	30.17(3.65)	30.55 ± 4.13	29.71 ± 2.89	0.001
SF-36 MH	50.06(3.38)	49.53 ± 3.75	50.69 ± 2.75	<0.001

HBCCS, home-based community care services; PHQ-9, The Chinese version of Patient Health Questionnaire; GAD-7, 7-item Generalized Anxiety Disorder; UCLA-3, University of California Los Angeles 3-item Loneliness Scale; SF-36 MH, 36-item Short-Form Health Survey mental health (MH) dimension.

### 3.2 Multivariate regression results

The results of the multiple linear regression for all respondents are presented in Table 2. After adjusting for sex, age, self-reported health status, number of diseases, self-care ability, marital status, monthly income, location, and education level, for every degree of satisfaction with medical care services, there was a 0.133 and 0.313 point decrease in the PHQ-9 and GAD-7 scores, respectively (*p* = 0.041 and *p* = 0.002, respectively). A higher level of social and recreational services satisfaction was associated with a lower UCLA-3 score (*β* = −0.266, *p* = 0.035). Furthermore, a higher level of spiritual comfort services satisfaction was significantly associated with lower PHQ-9 and UCLA-3 scores (*β* = −0.135, *p* = 0.042; *β* = −0.478, *p* < 0.001, respectively) and higher SF-36 MH scores (*β* = 0.565, *p* < 0.001).

**Table 2:** The association between HBCCS satisfaction and mental health scores among all respondents.

Variable	PHQ-9	GAD-7	UCLA-3	SF-36 MH
Sex (ref: male)	0.075(0.103)	0.040(0.158)	−0.166(0.201)	0.264(0.191)
Age	−0.003(0.006)	0.003(0.009)	−0.029(0.012)^*^	−0.016(0.011)
Self-reported health status	−0.189(0.072)^*^	−0.024(0.111)	−0.585(0.140)^**^	0.336(0.133)^*^
Number of disease (ref: 0)				
1–2	0.132(0.145)	−0.438(0.224)^*^	0.193(0.285)	0.528(0.270)
>2	0.366(0.145)^*^	−0.216(0.223)	0.684(0.284)^*^	0.285(0.269)
Self-care ability (ref: none)				
Partly	−2.346(0.358)^**^	−2.647(0.550)^**^	−3.688(0.702)^**^	3.085(0.663)^**^
Completely	−2.335(0.356)^**^	−2.620(0.547)^**^	−3.587(0.698)^**^	3.719(0.662)^**^
Marital status (ref: married/cohabitating)	0.663(0.275)^*^	0.713(0.424)	1.360(0.540)^*^	−0.872(0.512)
Monthly income (ref: <$155)				
$155–$310	−0.435(0.337)	−1.477(0.520)^**^	−0.297(0.661)	0.885(0.627)
$310–$465	−0.352(0.361)	−1.352(0.558)^*^	−0.264(0.710)	1.504(0.673)^*^
$465–$620	−0.651(0.350)	−1.356(0.540)^*^	−0.671(0.687)	1.081(0.651)
≥$620	−0.650(0.358)	−1.429(0.552)^*^	−0.733(0.703)	1.004(0.666)
Location (ref: urban)	−0.199(0.105)	−0.050(0.161)	−0.009(0.205)	0.491(0.195)^*^
Education (ref: junior high school and lower)	−0.085(0.104)	−0.316(0.160)	0.107(0.204)	0.190(0.193)
Satisfaction of community care services				
Daily care services	−0.126(0.066)	0.062(0.102)	−0.023(0.130)	0.067(0.123)
Medical care services	−0.133(0.065)^*^	−0.313(0.100)^**^	−0.176(0.128)	0.081(0.121)
Social and recreational services	0.104(0.064)	−0.168(0.099)	−0.266(0.126)^*^	0.023(0.119)
Spiritual comfort services	−0.135(0.066)^*^	−0.137(0.102)	−0.478(0.130)^**^	0.565(0.123)^**^
Adjusted *R*^2^	0.367	0.249	0.368	0.339

^*^p < 0.05, ^**^p < 0.01. HBCCS, home-based community care services; PHQ-9, The Chinese version of Patient Health Questionnaire; GAD-7, 7-item Generalized Anxiety Disorder; UCLA-3, University of California Los Angeles 3-item Loneliness Scale; SF-36 MH, 36-item Short-Form Health Survey mental health dimension.

Table 3 shows the association between mental health and HBCCS satisfaction in the urban and rural groups. Among the urban respondents, satisfaction with medical care services had a significantly negative relationship with PHQ-9 and UCLA-3 scores (*β* = −0.296, *p* = 0.004; *β* = −0.447, *p* = 0.009, respectively), satisfaction with social and recreational services had a significantly negative relationship with UCLA-3 scores (*β* = −0.426, *p* = 0.010), while satisfaction with spiritual comfort services had a significantly negative relationship with UCLA-3 scores (*β* = −0.523, *p* = 0.002) and a positive relationship with SF-36 MH component scores (*β* = 0.646, *p* < 0.001). The rural respondents showed a negative association between satisfaction with medical care services and GAD-7 scores (*β* = −0.327, *p* = 0.028).

**Table 3:** The association between HBCCS satisfaction and mental health scores among the urban and rural older adults.

Variable	PHQ-9	GAD-7	UCLA-3	SF-36 MH
Urban				
Satisfaction of community care services				
Daily care services	−0.130(0.100)	0.063(0.135)	−0.005(0.169)	−0.122(0.167)
Medical care services	−0.296(0.101)^**^	−0.244(0.136)	−0.447(0.171)^**^	−0.026(0.169)
Social and recreational services	0.138(0.098)	0.035(0.131)	−0.426(0.166)^*^	0.083(0.164)
Spiritual comfort services	−0.121(0.101)	−0.265(0.136)	−0.523(0.172)^**^	0.646(0.170)^**^
CV	Yes	Yes	Yes	Yes
Adjusted *R*^2^	0.450	0.398	0.544	0.461
Rural				
Satisfaction of community care services				
Daily care services	−0.033(0.081)	0.209(0.158)	0.111(0.199)	0.194(0.186)
Medical care services	0.048(0.076)	−0.327(0.149)^*^	0.189(0.187)	0.145(0.175)
Social and recreational services	0.132(0.077)	−0.270(0.152)	0.044(0.191)	−0.197(0.179)
Spiritual comfort services	−0.072(0.080)	0.132(0.158)	−0.130(0.198)	0.350(0.186)
CV	Yes	Yes	Yes	Yes
Adjusted *R*^2^	0.059	0.004	0.001	0.028

^*^p < 0.05, ^**^p < 0.01. HBCCS, home-based community care services; CV, control variables; PHQ-9, The Chinese version of Patient Health Questionnaire; GAD-7, 7-item Generalized Anxiety Disorder; UCLA-3, University of California Los Angeles 3-item Loneliness Scale; SF-36 MH, 36-item Short-Form Health Survey mental health dimension.

### 3.3 Robustness test results

Table 4 shows the linear regression models without the control variables. To ensure the reliability of the results in Tables 2, 3, we removed the control variables to test the robustness of the results, with only four community care service satisfaction items set as independent variables. The results indicated that the significance and direction of the coefficients of the dependent variables in Table 4 were consistent with those in Tables 2, 3, which suggested that the estimates and results concerning the association between HBCCS satisfaction and mental health in this study were robust and reliable.

**Table 4:** Linear regression models without the control variables.

Variable	PHQ-9	GAD-7	UCLA-3	SF-36 MH
Total				
Satisfaction of community care services				
Daily care services	−0.330(0.069)^**^	−0.123(0.102)	−0.363(0.133)^**^	0.306(0.126)^**^
Medical care services	−0.410(0.067)^**^	−0.604(0.098)^**^	−0.681(0.129)^**^	0.532(0.122)^**^
Social and recreational services	−0.114(0.066)	−0.401(0.098)^**^	−0.626(0.128)^**^	0.370(0.121)^*^
Spiritual comfort services	−0.406(0.067)^**^	−0.422(0.099)^**^	−0.967(0.129)^**^	1.025(0.122)^**^
CV	No	No	No	No
Adjusted *R*^2^	0.233	0.178	0.264	0.230
Urban				
Satisfaction of community care services				
Daily care services	−0.356(0.101)^**^	−0.208(0.136)	−0.363(0.173)^*^	0.206(0.173)
Medical care services	−0.651(0.098)^**^	−0.710(0.132)^**^	−1.038(0.169)^**^	0.616(0.167)^**^
Social and recreational services	−0.128(0.097)	−0.342(0.130)^**^	−0.802(0.165)^**^	0.520(0.164)^**^
Spiritual comfort services	−0.447(0.098)^**^	−0.607(0.131)^**^	−1.140(0.167)^**^	1.242(0.166)^**^
CV	No	No	No	No
Adjusted *R*^2^	0.361	0.303	0.462	0.354
Rural				
Satisfaction of community care services				
Daily care services	−0.099(0.079)	0.186(0.150)	0.006(0.191)	0.206(0.179)
Medical care services	0.054(0.076)	−0.302(0.144)^*^	0.122(0.182)	0.224(0.171)
Social and recreational services	0.108(0.077)	−0.294(0.146)^*^	−0.011(0.185)	−0.114(0.174)
Spiritual comfort services	−0.055(0.081)	0.101(0.153)	−0.165(0.194)	0.346(0.183)
CV	No	No	No	No
Adjusted *R*^2^	0.001	0.015	−0.008	0.011

^*^p < 0.05, ^**^p < 0.01. The results were presented as β (Standard Error). HBCCS, home-based community care services; CV, control variables; PHQ-9, The Chinese version of Patient Health Questionnaire; GAD-7, 7-item Generalized Anxiety Disorder; UCLA-3, University of California Los Angeles 3-item Loneliness Scale; SF-36 MH, 36-item Short-Form Health Survey mental health dimension.

## 4 Discussion

In this study, we investigated the associations between four types of HBCCS satisfaction and the mental health level of older individuals, including the differences in these associations among urban–rural groups. Data analysis revealed that HBCCS satisfaction is a significant factor likely to influence the mental health status among older adults in Zhejiang Province. The results of this study showed that, in general, satisfaction with HBCCS among the older rural adults was significantly higher than that among those living in urban areas. This finding is inconsistent with previous studies (4). There are three possible reasons for this finding. First, in response to the disparity in satisfaction with HBCCS between rural and urban areas, the Zhejiang Provincial government has expanded investment in welfare institutions and facilities, making HBCCS more accessible to older people in rural villages (5). Extending the concept of socialization to HBCCS and making full use of services provided by families, communities, and institutions are essential to meet the growing needs of older adults in rural areas. Second, previous studies have demonstrated that the proportion of those willing to engage with HBCCS is greater in rural older adults than in urban older adults (32), and a higher level of willingness might drive higher satisfaction with HBCCS (33). Third, the proportion of older adults in urban areas with high educational levels was greater than that of their rural counterparts. A previous study demonstrated that older adults with high educational levels expected higher HBCCS quality (34), which may lead to relatively lower HBCCS satisfaction than their rural counterparts.

Interestingly, this study found that the mental health of the rural older adults was significantly better than that of the urban older adults, which is inconsistent with the findings of Sun and Lyu (19). They found that rural older adults had worse mental health than their urban counterparts using the data sourced from the 2014 wave of the Chinese Longitudinal Healthy Longevity Survey (CLHLS) (19). This inconsistency might be explained by the small rural–urban difference in socio-economic status in Zhejiang Province, given that socio-economic status is the major factor resulting in rural–urban mental health problems divergence in China (35). However, the findings are consistent with those of relevant studies conducted in developed countries. One reason for these findings may be that urban life in developed areas, including Zhejiang Province in this study, may be more stressful relative to rural living due to more crowded environments, higher pollution level, and a faster pace (36). Additionally, in this study, the rural older adults reported better self-reported health status and fewer diseases than their rural counterparts. Physical illnesses and disabilities are known to increase the risk of mental health problems in older adults (37).

The key findings of this study indicate that in general, HBCCS satisfaction had a positive relationship with mental health among the older adults, which is similar to the findings of many previous studies conducted in China. Wang et al. (38) found that using home and community care services decreased depression scores among older adults. Another study demonstrated that community-based support and services were significantly associated with a good quality of life among older adults in China (39), and a high quality of life was found to be positively associated with better mental health status (40). A study conducted in western China also found that the utilization of daily care services and social and recreational services was significantly and positively associated with good mental health in older adults (4).

Higher satisfaction levels with social and recreational services were positively associated with mental health among the urban respondents, which was similar to the findings of Yang et al. (4), who conducted their study in Shanxi Province. Although the types of social and recreational services in Zhejiang Province and Shanxi Province may differ, such social activities would appear to be effective in alleviating the symptoms of mental health problems among older Chinese adults living in urban areas. Previous studies have also indicated that participation in social activities plays a greater role in relieving mental health symptoms in urban older adults than in rural older adults (41).

The results also indicated that satisfaction with medical care services was significantly and negatively associated with GAD-7 scores among the rural older adults, whereas other services had no significant association with mental health status. Similarly, Chen and Hao (42) found that providing home-based healthcare had a significantly positive impact on mental health, whereas providing spiritual comfort services did not. Several medical illnesses are highly comorbid with anxiety disorders in older adults (43); as a result, medical services may improve anxiety levels. Nevertheless, social and recreational services among rural Chinese older adults mainly organized by the rural community, and spiritual comfort services in rural areas in China mainly refer to non-professional psychological counseling services. Therefore, the professional level of these services provided by the rural community is likely to fall far short of meeting the psychological needs of rural older adults and in improving their GAD-7 scores (12).

In order to improve HBCCS satisfaction and alleviate mental health problems among this vulnerable population, several targeted interventions are recommended. First, health policy makers should collaborate to promote HBCCS facilities with qualified personnel for older adults. Second, the service quality of HBCCS needs to be improved for older adults, especially medical care services, social and recreational services, spiritual comfort services for urban older adults, and medical care services for rural older adults. Finally, higher vocational colleges in Zhejiang Province should train the relevant professionals to provide high-quality HBCCS.

This study has some significant strengths. First, to the best of our knowledge, this is the first study to provide evidence of an association between HBCCS satisfaction and mental health status among older adults in Zhejiang Province. The participants included in this study were representative of Zhejiang Province; thus, the findings could be generalized to the entire Zhejiang Province. Second, confounding biases were decreased through using multi-variable adjustment. Third, we estimated mental health status using previously developed measurement tools that have shown good reliability and validity.

This study has some limitations. First, because of the nature of this cross-sectional study, we could not determine causal relationships. Second, assessing mental health among the older adult population is a complex issue. Other factors associated with mental health among older adults were not controlled for in this study, which may have biased the results. Third, the mental health questionnaires provided self-reported detail on mental health and no objective measures of mental health were used; therefore, reporting bias is a concern. Finally, the survey was conducted only in Zhejiang Province, which has one of the highest levels of economic development in China. Thus, a further cohort study should be conducted using diagnostic mental health criteria and controlling for more factors associated with mental health among older adults. In addition, in future studies, more provinces that better represent the overall economic development level in China should be included to explore differences between regions with different economic levels.

## 5 Conclusion

In this cross-sectional study, we found a strong correlation between high HBCCS satisfaction and better mental health status among older adults in Zhejiang Province, with the older adults in rural areas rating HBCCS more highly. These findings could help inform the development of more effective HBCCS strategies to continuously improve satisfaction with HBCCS in Zhejiang Province, especially to maintain HBCCS satisfaction that is relevant to the mental health of rural and urban older adults.

## Data Availability

The raw data supporting the conclusions of this article will be made available by the authors, without undue reservation.

## References

[1] The Lancet. Population ageing in China: crisis or opportunity? Lancet. (2022) 400:1821. doi: 10.1016/S0140-6736(22)02410-2 36436518

[2] Chan AKY Tamrakar M Jiang CM Lo ECM Leung KCM Chu CH. Common medical and dental problems of older adults: a narrative review. Geriatrics. (2021) 6:76. doi: 10.3390/geriatrics6030076, PMID: 34449647 PMC8395714

[3] Zhang Y Liu X Meng Q Li B Caneparo L. Physical environment research of the family ward for a healthy residential environment. Front Public Health. (2022) 10:1015718. doi: 10.3389/fpubh.2022.1015718, PMID: 36311645 PMC9606755

[4] Yang L Wang L Dai X. Rural-urban and gender differences in the association between community care services and elderly individuals' mental health: a case from Shaanxi Province, China. BMC Health Services Res. (2021) 21:106. doi: 10.1186/s12913-021-06113-z, PMID: 33516212 PMC7847576

[5] Zhou W Jiang B Yu L Dai W. Measuring demand and supply of community care services for older people healthy ageing in rural Zhejiang Province. BMC Geriatr. (2022) 22:286. doi: 10.1186/s12877-022-02906-x, PMID: 35387605 PMC8985291

[6] Zhang J Sun X Yao A. Use of home and community-based services and loneliness in older people with functional limitations: a cross-sectional study. BMC Psychiatry. (2023) 23:717. doi: 10.1186/s12888-023-05225-6, PMID: 37794343 PMC10548717

[7] Bao J Zhou L Liu G Tang J Lu X Cheng C. Current state of care for the elderly in China in the context of an aging population. Biosci Trends. (2022) 16:107–18. doi: 10.5582/bst.2022.01068 35431289

[8] Hu B Li B Wang J Shi C. Home and community care for older people in urban China: receipt of services and sources of payment. Health Social Care Community. (2020) 28:225–35. doi: 10.1111/hsc.12856, PMID: 31508864

[9] Lu P Shelley M Kong D. Unmet community service needs and life satisfaction among Chinese older adults: a longitudinal study. Soc Work Pub Health. (2021) 36:665–76. doi: 10.1080/19371918.2021.1948942, PMID: 34340642

[10] Zeng W Zhao P Zhao Y Saddique R. The multidimensional relative poverty of rural older adults in China and the effect of the health poverty alleviation policy. Front Public Health. (2022) 10:793673. doi: 10.3389/fpubh.2022.793673, PMID: 35937214 PMC9354235

[11] Ke Y Jiang J Chen Y. Social capital and the health of left-behind older adults in rural China: a cross-sectional study. BMJ Open. (2019) 9:e030804. doi: 10.1136/bmjopen-2019-030804, PMID: 31772090 PMC6886947

[12] Liu ZW Yu Y Fang L Hu M Zhou L Xiao SY. Willingness to receive institutional and community-based eldercare among the rural elderly in China. PLoS One. (2019) 14:e0225314. doi: 10.1371/journal.pone.0225314, PMID: 31756228 PMC6874313

[13] Qi H Shen X Long F Liu M Gao X. Spatial-temporal characteristics and influencing factors of county-level carbon emissions in Zhejiang Province, China. Environ Sci Pollut Res Int. (2023) 30:10136–48. doi: 10.1007/s11356-022-22790-7, PMID: 36070039

[14] The State Council of the People’s Republic of China. In order to explore a path towards promoting common prosperity for all people, we visited relevant officials of the National Development and reform commission to discuss their support for Zhejiang's high-quality development and the establishment of a demonstration zone for common prosperity. (2021). Available at: https://www.gov.cn/zhengce/2021-06/10/content_5616869.htm/ (accessed June 15, 2024)

[15] Hu T Zhao X Wu M Li Z Luo L Yang C. Prevalence of depression in older adults: a systematic review and meta-analysis. Psychiatry Res. (2022) 311:114511. doi: 10.1016/j.psychres.2022.114511 35316691

[16] Lu L Wang SB Rao W Zhang Q Ungvari GS Ng CH. The prevalence of sleep disturbances and sleep quality in older Chinese adults: a comprehensive meta-analysis. Behavi Sleep Med. (2019) 17:683–97. doi: 10.1080/15402002.2018.1469492, PMID: 29851516

[17] Deng Y Zhao S Cheng G Yang J Li B Xu K. The prevalence of mild cognitive impairment among Chinese people: a meta-analysis. Neuroepidemiology. (2021) 55:79–91. doi: 10.1159/000512597, PMID: 33756479

[18] Yaka E Keskinoglu P Ucku R Yener GG Tunca Z. Prevalence and risk factors of depression among community dwelling elderly. Arch Gerontol Geriatr. (2014) 59:150–4. doi: 10.1016/j.archger.2014.03.014 24767692

[19] Sun J Lyu S. Social participation and urban-rural disparity in mental health among older adults in China. J Affect Disord. (2020) 274:399–404. doi: 10.1016/j.jad.2020.05.091, PMID: 32663969

[20] Niu L Jia C Ma Z Wang G Yu Z Zhou L. The validity of proxy-based data on loneliness in suicide research: a case-control psychological autopsy study in rural China. BMC Psychiatry. (2018) 18:116. doi: 10.1186/s12888-018-1687-x, PMID: 29716552 PMC5930838

[21] Jacob ME Abraham VJ Abraham S Jacob KS. The effect of community based daycare on mental health and quality of life of elderly in rural South India: a community intervention study. Int J Geriatr Psychiatry. (2007) 22:445–7. doi: 10.1002/gps.1706, PMID: 17096463

[22] Djernes JK. Prevalence and predictors of depression in populations of elderly: a review. Acta Psychiatr Scand. (2006) 113:372–87. doi: 10.1111/j.1600-0447.2006.00770.x 16603029

[23] Singh AS Masuku MB. Sampling techniques and determination of sample size in applied statistics research: an overview. Int J Econ Commer Manage. (2014) 2:1–22.

[24] Kroenke K Spitzer RL Williams JB. The PHQ-9: validity of a brief depression severity measure. J General Intern Med. (2001) 16:606–13. doi: 10.1046/j.1525-1497.2001.016009606.x, PMID: 11556941 PMC1495268

[25] Wang W Bian Q Zhao Y Li X Wang W Du J. Reliability and validity of the Chinese version of the patient health questionnaire (PHQ-9) in the general population. Gen Hospital Psychiatry. (2014) 36:539–44. doi: 10.1016/j.genhosppsych.2014.05.021, PMID: 25023953

[26] Spitzer RL Kroenke K Williams JB Löwe B. A brief measure for assessing generalized anxiety disorder: the GAD-7. Arch Intern Med. (2006) 166:1092–7. doi: 10.1001/archinte.166.10.1092 16717171

[27] Yu W Singh SS Calhoun S Zhang H Zhao X Yang F. Generalized anxiety disorder in urban China: prevalence, awareness, and disease burden. J Affect Disord. (2018) 234:89–96. doi: 10.1016/j.jad.2018.02.012, PMID: 29524751

[28] Russell DW. UCLA loneliness scale (version 3): reliability, validity, and factor structure. J Personality Assess. (1996) 66:20–40. doi: 10.1207/s15327752jpa6601_2, PMID: 8576833

[29] Liu T Lu S Leung DKY Sze LCY Kwok WW Tang JYM. Adapting the UCLA 3-item loneliness scale for community-based depressive symptoms screening interview among older Chinese: a cross-sectional study. BMJ Open. (2020) 10:e041921. doi: 10.1136/bmjopen-2020-041921 PMC7733209 33303463

[30] McHorney CA Ware JE Jr Raczek AE. The MOS 36-item short-form health survey (SF-36): II. Psychometric and clinical tests of validity in measuring physical and mental health constructs. Med Care. (1993) 31:247–63. doi: 10.1097/00005650-199303000-00006 8450681

[31] Hu J Gruber KJ Hsueh KH. Psychometric properties of the Chinese version of the SF-36 in older adults with diabetes in Beijing, China. Diabetes Res Clin Pract. (2010) 88:273–81. doi: 10.1016/j.diabres.2010.03.005, PMID: 20392508

[32] Xia Y Xu L Sun L Li J Qin W Zhang J. Rural-urban differences in home-based care willingness among older adults: a cross-sectional study in Shandong, China. Int J Qual Health Care. (2020) 32:126–34. doi: 10.1093/intqhc/mzz132, PMID: 32242222

[33] Shi L Patil VP Leung W Zheng Q. Willingness to use and satisfaction of primary care services among locals and migrants in Shenzhen, China. Health Soci Care Community. (2022) 30:e113–25. doi: 10.1111/hsc.13418, PMID: 33978287

[34] Ma W Wang J Liu L Zhang H. Factors influencing the satisfaction of community senior care services in China: an analysis based on an extended Anderson model. Front Public Health. (2023) 11:1138711. doi: 10.3389/fpubh.2023.1138711, PMID: 37427287 PMC10326384

[35] Li LW Liu J Xu H Zhang Z. Understanding rural-urban differences in depressive symptoms among older adults in China. J Aging Health. (2016) 28:341–62. doi: 10.1177/0898264315591003, PMID: 26100620 PMC4893784

[36] Melis G Gelormino E Marra G Ferracin E Costa G. The effects of the urban built environment on mental health: a cohort study in a large northern Italian city. Int J Environ Res Public Health. (2015) 12:14898–915. doi: 10.3390/ijerph121114898, PMID: 26610540 PMC4661687

[37] Reynolds CF 3rd Jeste DV Sachdev PS Blazer DG. Mental health care for older adults: recent advances and new directions in clinical practice and research. World Psychiatry. (2022) 21:336–63. doi: 10.1002/wps.20996, PMID: 36073714 PMC9453913

[38] Wang Q Fan K Li P. Effect of the use of home and community care services on the multidimensional health of older adults. Int J Environ Res Public Health. (2022) 19:15402. doi: 10.3390/ijerph192215402, PMID: 36430119 PMC9690765

[39] Zhang Y Yeager VA Hou S. The impact of community-based supports and services on quality of life among the elderly in China: a longitudinal study. J Appl Gerontol. (2018) 37:1244–69. doi: 10.1177/0733464816661945, PMID: 27496140 PMC7091461

[40] Bai W Zhang J Smith RD Cheung T Su Z Ng CH. Inter-relationship between cognitive performance and depressive symptoms and their association with quality of life in older adults: a network analysis based on the 2017-2018 wave of Chinese longitudinal healthy longevity survey (CLHLS). J Affect Disord. (2023) 320:621–7. doi: 10.1016/j.jad.2022.09.159, PMID: 36206885

[41] Guo Q Bai X Feng N. Social participation and depressive symptoms among Chinese older adults: a study on rural-urban differences. J Affect Disord. (2018) 239:124–30. doi: 10.1016/j.jad.2018.06.036, PMID: 30005325

[42] Chen QQ Hao Y. A study on the effect of community old-age service on the mental health improvement of the elderly. Northwest Pop J. (2020) 41:79–91. doi: 10.15884/j.cnki.issn.1007-067

[43] Wolitzky-Taylor KB Castriotta N Lenze EJ Stanley MA Craske MG. Anxiety disorders in older adults: a comprehensive review. Depress Anxiety. (2010) 27:190–211. doi: 10.1002/da.20653 20099273

[44] Li H Jia J Yang Z. Mini-mental state examination in elderly Chinese: a population-based normative study. J Alzheimers Dis. (2016) 53:487–96. doi: 10.3233/JAD-160119, PMID: 27163822

